# BERT-Based Approaches to Identifying Malicious URLs

**DOI:** 10.3390/s23208499

**Published:** 2023-10-16

**Authors:** Ming-Yang Su, Kuan-Lin Su

**Affiliations:** Department of Computer Science and Information Engineering, Ming Chuan University, Taoyuan City 333, Taiwan; qpaqpa48@gmail.com

**Keywords:** malicious URL, phishing, BERT, IoT, DoH

## Abstract

Malicious uniform resource locators (URLs) are prevalent in cyberattacks, particularly in phishing attempts aimed at stealing sensitive information or distributing malware. Therefore, it is of paramount importance to accurately detect malicious URLs. Prior research has explored the use of deep-learning models to identify malicious URLs, using the segmentation of URL strings into character-level or word-level tokens, and embedding and employing trained models to differentiate between URLs. In this study, a bidirectional encoder representation from a transformers-based (BERT) model was devised to tokenize URL strings, employing its self-attention mechanism to enhance the understanding of correlations among tokens. Subsequently, a classifier was employed to determine whether a given URL was malicious. In evaluating the proposed methods, three different types of public datasets were utilized: a dataset consisting solely of URL strings from Kaggle, a dataset containing only URL features from GitHub, and a dataset including both types of data from the University of New Brunswick, namely, ISCX 2016. The proposed system achieved accuracy rates of 98.78%, 96.71%, and 99.98% on the three datasets, respectively. Additionally, experiments were conducted on two datasets from different domains—the Internet of Things (IoT) and Domain Name System over HTTPS (DoH)—to demonstrate the versatility of the proposed model.

## 1. Introduction

In July 2022, the Interisle Consulting Group published a report on the phishing landscape covering the period from 1 May 2021 to 30 April 2022 [1]. The report highlighted that over 3 million phishing events were detected, resulting in 1.1 million unique phishing attacks during this period. Compared to a previous annual report released in July 2021, the number of unique phishing attacks increased by 61%. Additionally, there was a 72% rise in malicious domain names and an 83% increase in registered domain names used by phishers. Furthermore, cryptocurrency phishing attacks experienced a significant surge of 257%, explicitly targeting digital currency wallets and exchanges. According to Trend Micro’s 2022 Cyber Security Report [2], over 16 million phishing attempts were detected in 2021 worldwide, a 2.3-fold increase from the previous year. Of these incidents, 62% originated from spam and 38% were associated with fake login pages. Furthermore, 90% of data breaches in 2021 were attributed to phishing emails. The report further highlighted the increasing vulnerability of non-fungible tokens (NFTs) to fraud, with scams involving fake NFT exchange domains and deceptive websites that trick users into linking their wallets while facilitating subsequent attacks.

URL analysis typically involves feature extraction or character embedding. Feature extraction identifies essential URL attributes, such as domain, length, and character count, for input into classification algorithms. Early machine learning models [3,4,5,6,7,8] relied on manual feature extraction for accuracy. However, they were limited by their reliance on historical data and time-consuming pre-processing, reducing the effectiveness of their real-time cyberattack defense. Character embedding converts individual URL characters into vectors, enabling deep-learning models to assess URL maliciousness. However, it has limitations, including different meanings for the same character in various URL positions and a lack of character relationship consideration. In addressing this gap, ensemble models [9,10,11,12,13] have often been used alongside character embeddings to capture URL features before making decisions. Nevertheless, these malicious URL detection models were primarily tailored for datasets with URL strings that lack the versatility to handle different data formats or domains effectively.

Therefore, to overcome these limitations, this paper introduces a BERT-based (bidirectional encoder representations from transformers) [14] approach to enhance the detection of malicious URLs. This model excels at effectively capturing semantic relationships. In particular, this study conducts a comprehensive evaluation using various publicly available datasets, including those from Kaggle, GitHub, and ISCX 2016, to ensure a rigorous and robust analysis. The proposed model achieves remarkably high accuracy rates across all the datasets. Furthermore, the study assesses the model’s performance on both direct URL strings and their derived feature representations, showcasing its versatility. Additionally, the research extends the model’s applicability beyond detecting malicious URLs to include attacks in IoT and DoH domains, highlighting its flexibility.

The remaining sections of this paper are organized as follows: Section 2 offers an overview of related research and highlights the contributions of this study. Section 3 details the proposed approaches for URL analysis. Section 4 presents the experimental results on a variety of datasets, including accuracy, extensibility, and prediction time requirements. Lastly, Section 5 provides a summary of the advantages, performance, and limitations of the proposed system.

## 2. Related Research

Kumar et al. [3] proposed a multi-layer model for the detection of malicious URLs. This model comprises several filters, including a stratified filter, a Naïve Bayesian filter, a CART decision tree filter, and a Support Vector Machine (SVM) filter. Each filter makes its unique contribution, resulting in an overall model accuracy of 79.55%. Ahammad et al. [4] proposed a machine learning-based phishing detection method that extracts 15 features from a URL. These features include the domain name, URL length, depth, domain name system (DNS) records, presence in PhishTank, connection traffic, and other factors. Various machine learning classifiers, including decision trees, random forests, Light GBM, and support vector machines, were used with these features as inputs. Their findings indicate that Light GBM achieved the highest accuracy rate of 86%. In contrast, Gupta et al. [5] introduced a streamlined approach utilizing nine lexical features for phishing detection, including the number of tokens in domain names, the number of top-level domains, URL length, domain length, and the number of separators. By employing a random forest classifier, they achieved an accuracy of 99.57%. Saleem et al. [6] developed a lightweight machine-learning method for detecting malicious URLs. They selected 27 lexical features, removed 7 irrelevant ones, and trained a classifier using the remaining 20 features. Their results demonstrate that random forest achieved 99% accuracy, while k-nearest neighbors (KNNs) achieved 98% accuracy, with a reduced processing time. The authors suggested that KNN offers a better balance between time and accuracy, making it a preferred choice.

Li et al. [7] emphasized the importance of feature engineering. They introduced five linear and nonlinear space transformation methods to address the challenges faced by traditional classifiers. Using 62 features, the authors found that the experimental results exhibited notable enhancements in the accuracy of KNN, SVM, and multi-layer perceptron (MLP). The correct rates increased from 68%, 58%, and 63% to 86%, 81%, and 82%, respectively. Mondal et al. [8] introduced a framework named SeizeMaliciousURL that utilizes an ensemble classifier for identifying malicious URLs through voting. By leveraging the distinctive strengths of multiple classifiers, the framework aggregates their probabilities to make a final decision. Their results demonstrated that SeizeMaliciousURL outperformed individual machine learning methods, achieving superior outcomes. Amid the heightened phishing threats during the COVID-19 pandemic, Piñeiro et al. [15] proposed a web architecture integrating three machine-learning algorithms: random forest, classification tree, and support vector machine. By combining these models’ outputs through operations, their system aimed for enhanced identification. Results showed that the pure classification tree achieved the highest prediction accuracy at 80%. Kalabarige et al. [16] introduced MLSELM, a multi-layer stacking integrated learning technology with three layers. The first layer includes five classifiers: KNN, MLP, extreme gradient boosting (XGB), random forest (RF), and logistic regression (LR). The second layer selects the top three classifiers from the first layer, and their outputs proceed to the next layer. Subsequently, a “meta-learner” predicts whether a URL is phishing. Experimental findings across three datasets (D1, D2, D3, and D4) revealed that MLSELM attained accuracies of 97.76%, 98.90%, 96.79%, and 98.43%, respectively, slightly surpassing individual basic models. Somesha et al. [17] proposed a method for classifying phishing and regular emails based on headers. They utilized word embedding, term frequency-inverse document frequency (TF-IDF), and FastText for feature extraction and employed machine learning algorithms, like RF, SVM, LR, XGB, and decision tree (DT). Results showed that RF achieved the highest accuracy of 99.5%, coupled with a low false alarm rate of 0.053% when paired with FastText.

Furthermore, certain studies employed deep-learning models to address the issue of classifying URLs. Li et al. [18] proposed a long short-term memory (LSTM)-based method for detecting phishing emails, considering the increasing complexity of phishing camouflage. They combined KNN and K-Means to enhance the training dataset while achieving an accuracy of 95%. Srinivasan et al. [9] introduced DURLD, a method that converts URLs into character-level sequences and utilizes five models based on a convolutional neural network (CNN) and recurrent neural network (RNN) for identifying malicious URLs. Across multiple datasets, experiments yielded accuracies ranging from 93% to 98%. In particular, the authors highlighted the shorter training time required in comparison to traditional feature engineering-based methods. Bozkir et al. [10] introduced GramBeddings, a deep neural network that fuses a CNN with bidirectional long short-term memory (BiLSTM) and integrates a self-attention layer for phishing classification. L2 regularization was applied during training to curb overfitting, resulting in an accuracy of 98.27%. Singh et al. [19] employed GloVe embedding for pre-processing URLs and utilized a CNN-based model for phishing detection, achieving a notable accuracy of 98.00%. Ariyadasa et al. [20] introduced PhishDet, a phishing detection system that combines LSTM-CNN with URL and HTML features. By incorporating graph neural networks (GNNs), PhishDet reached an accuracy rate of 96.42%. The system comprises two independent models, URLDet and HTMLDet, for processing URL and HTML content, respectively. However, the authors stressed the importance of regular retraining to ensure consistent and sustained performance over time.

Alsaedi et al. [21] highlighted the limitations of relying solely on website content for phishing detection due to obfuscation techniques. They proposed CTI-MURLD, a two-stage integrated learning model incorporating network threat intelligence from Google searches and Whois websites to enhance detection performance. This model combines the random forest algorithm with an MLP classifier, leveraging the decision tree output as input for the MLP. Compared to traditional URL-based models, CTI-MURLD achieved a 7.8% accuracy increase and reduced the false positive rate by 6.7%. Alshehri et al. [11] proposed a lightweight deep-learning model for phishing detection. Using character-level embeddings to convert URLs into vector representations and utilizing merged CNN1D models, their approach achieved an accuracy of 98.13% while maintaining low computational resource requirements. The model also reduced execution time by up to 30% compared to word-level embeddings. Zheng et al. [12] proposed a deep convolutional neural network model (HDP-CNN) to tackle the reliance on expert features in phishing detection. By combining character- and word-level information and utilizing a deep pyramid structure, the model captures both the local and global features of URLs, achieving an impressive 98.3% accuracy. Hussain et al. [13] proposed a lightweight CNN-fusion model that utilized multiple CNNs with varying kernel sizes to extract features from URLs at different levels. This model achieved an accuracy of 99%. Notably, it is well-suited for devices with limited GPU memory. Remmide et al. [22] introduced a temporal convolutional network (TCN) with word embeddings, combining RNN and CNN to capture temporal and spatial features. Their results demonstrated an accuracy of 98.95%, accompanied by a precision, recall, and F1-score all reaching 98%. Wang et al. [23] introduced the TCURL hybrid network architecture, which combines CNNs and transformers to leverage CNNs’ positional information for replacing positional encoding in transformers. The self-attention mechanism captures internal dependencies, and the integrated output is utilized for binary classification. Experimental results on the ISCX 2016 dataset demonstrated an accuracy of 99.7%.

Maneriker et al. [24] introduced URLTran, which leverages advancements in natural language processing (NLP) to detect phishing URLs. URLTran comprises three distinct models: URLTran_RoBERTs, URLTran_BERT, and URLTran_CustVoc. The former two models employ word piece and byte pair encoding (BPE) techniques for tokenization, while the latter utilizes customized character-level BPE vocabularies derived from the training dataset. The authors also utilized two other models, URLNet and Texception, as baselines to show the performances of URLTran. The experiments were conducted using a non-public dataset. The URLTran_BERT model outperformed the others slightly, achieving an accuracy of 99.67%. The authors noted that when setting the false positive rate (FPR) to 0.01%, URLTran BERT attained a true positive rate (TPR) of 86.80%, surpassing URLNet at 71.20% and Texception at 52.15%. It is worth noting that all URLTran models exclusively accepted URL strings as input. Ullah et al. [25] harnessed BERT to construct an interpretable malware detection system tailored for the Android platform. They utilized BERT to extract the acquired textual features and subsequently introduced an algorithm to convert malware into images, streamlining the transformation of network byte streams into visual representations. Lin et al. [26] introduced ET-BERT, a model for classifying encrypted network traffic. It employs pre-training to develop deep contextualized datagram-level representations from extensive unlabeled data. With fine-tuning on a limited amount of task-specific labeled data, ET-BERT achieved excellent performance across five encrypted traffic classification tasks. Shi et al. [27] introduced a BERT-based time-series feature network (TSFN) model designed for the identification of malicious traffic. This model consists of two key components. The first part utilizes BERT to capture global features of the traffic, while the second part employs LSTM to capture the time-series characteristics of the traffic. These two sets of features are then combined to represent the traffic effectively. Experiments conducted on the publicly available USTC-TFC dataset demonstrated that the model can achieve an impressive F1-score value of 99.50%.

In recent years, deep learning models have increasingly been considered replacements for traditional machine learning algorithms in addressing a wide range of security issues. This paper introduces a novel approach that makes the following contributions:

1. Utilization of BERT-based model with public datasets: The proposed system leverages three public datasets to demonstrate the effectiveness of the BERT-based model in detecting malicious URLs.
2. Feature-based detection: Even when provided with only URL features rather than the URL strings, the proposed method works well, highlighting the versatility of the approach in different scenarios.
3. Extensibility: The proposed method has the potential for extension to identify attacks in other environments, such as the Internet of Things (IoT) and Domain Name System over HTTPS (DoH), not solely limited to malicious URL detection.
4. Real-time detection: The proposed system is designed for real-time deployment, allowing it to swiftly detect malicious URLs as they appear, enhancing online security measures.

## 3. The Proposed Methodology

**Figure 1:**
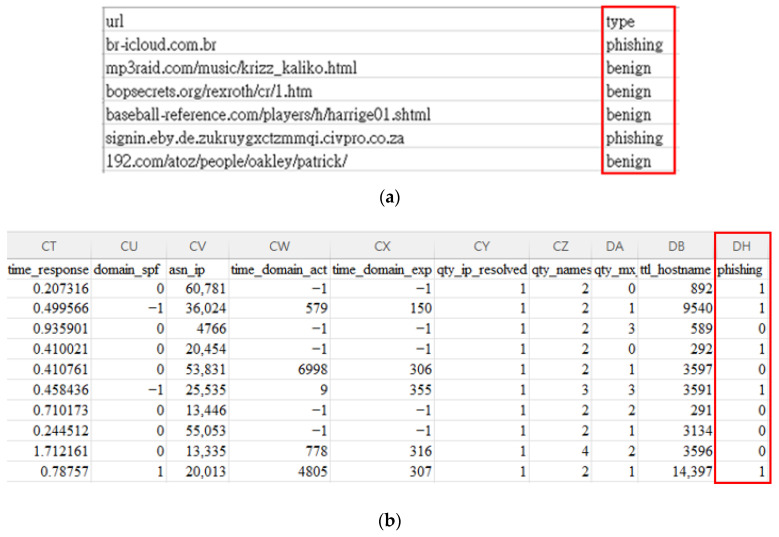
Different types of URL datasets. (**a**) URL strings and labels (https://www.kaggle.com/datasets/sid321axn/malicious-urls-dataset) (accessed on 26 August 2023). (**b**) URL features and labels (without URL string) (https://github.com/GregaVrbancic/Phishing-Dataset) (accessed on 26 August 2023).

Public datasets on URLs can be categorized into two types. One provides only the URL string (Figure 1a), like those on Kaggle [28], and the other offers various features extracted from the URLs (Figure 1b), seen in datasets on GitHub [29], with 111 features per URL. Some datasets, such as ISCX 2016 [30], include both URL strings and features.

**Figure 2:**
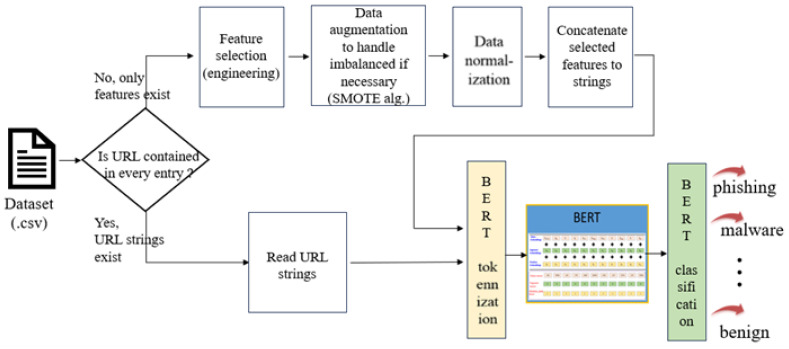
The proposed approach.

The approach in Figure 2 was designed to manage both types of datasets. When dealing with datasets containing only URL strings, BERT was utilized for tokenization, leveraging its self-attention mechanism to grasp semantic meaning. A subsequent classifier determined the maliciousness of each URL. For datasets comprising URL features without URL strings, the feature engineering algorithm—random forest—was used to select key features and form a feature string for each URL entry. Subsequently, a similar BERT process was applied to the feature strings of all entries. Essentially, the proposed system employed BERT for both URL strings and feature strings, facilitating the effective analysis and classification of malicious URLs. The algorithm is illustrated in Algorithm 1.
**Algorithm 1:** URL Classification**Input:** A dataset of labeled URLs or features, divided into 80% training and 20% testing. **Output:** Confusion matrix.1.    If the dataset contains URLs, use the training dataset to fine-tune the pre-trained BERT model and use the test dataset to evaluate.2.    Else://for the dataset with only features.3.         Select k important features using the random forest algorithm.4.         If the dataset is imbalanced, apply the SMOTE algorithm [31] to balance classes with fewer instances.5.         Normalize the data.6.         Concatenate selected features of each entry into a feature string with “/” as a separator.7.         Fine-tune the pre-trained BERT model using the training dataset.8.         Evaluate the model using the testing dataset.9.         Output the confusion matrix accompanied by accuracy, precision, and recall rate.10.  Endif

### 3.1. Data Pre-Processing

This section outlines the distinct pre-processing steps for the two types of datasets.

#### 3.1.1. URL String and BERT Tokenization

**Figure 3:**
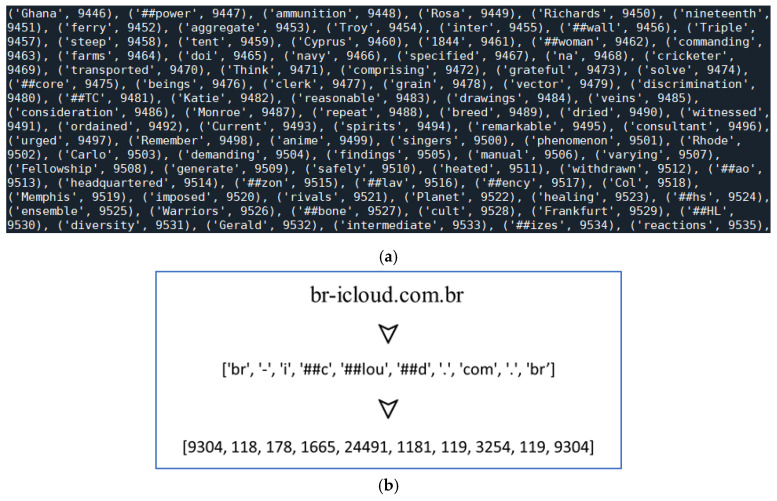
Illustration of BERT tokenization for an example URL string. (**a**) Part of the BERT dictionary and (**b**) BERT tokenization for the URL string.

For datasets containing only URL strings and labels, URL strings were used as the inputs for BERT tokenization. Unlike traditional character-level tokenization that assigns the same embeddings to identical characters, BERT tokenization takes into account the significance of letters within different vocabularies. In this study, the bert-base-cased model was employed for tokenizing the URL strings. The BERT token dictionary (see Figure 3a) and an example of URL tokenization (see Figure 3b) are provided. For example, the URL string “br-icloud.com.br” is divided into 10 tokens.

#### 3.1.2. URL Feature String and BERT Tokenization

**Figure 4:**
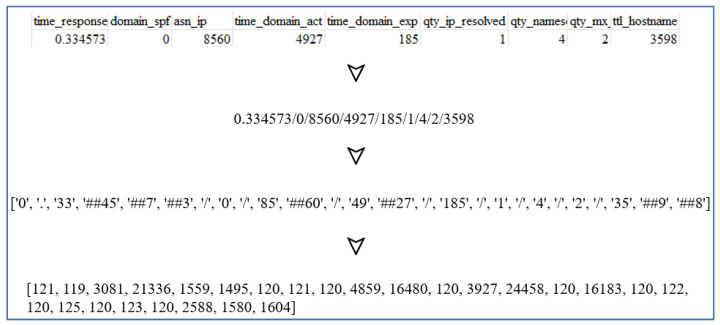
Illustration of BERT tokenization for URL features.

The pre-processing complexity increases when a dataset incorporates URL-related features without including URL strings. In the GitHub dataset [29], each entry is associated with 111 features and a label. This study selected essential features using the random forest algorithm and combined them into a feature string representation of the entry. To preserve feature integrity and prevent arbitrary tokenization during BERT processing, a separator “/” was added between essential features, as illustrated in Figure 4. For example, when two adjacent features have values of 25 and 24, the “/” separator results in the feature string “25/24” being used and split into three tokens: {“25”, “/”, and “24”}. Without the separator, “2524” could be split into tokens like {“2524”}, {“252” and “4”}, or {“2” and “524”}. The “/” separator helps maintain the integrity and distinction of individual features during tokenization.

### 3.2. Fine-Tuning the BERT Model

**Figure 5:**
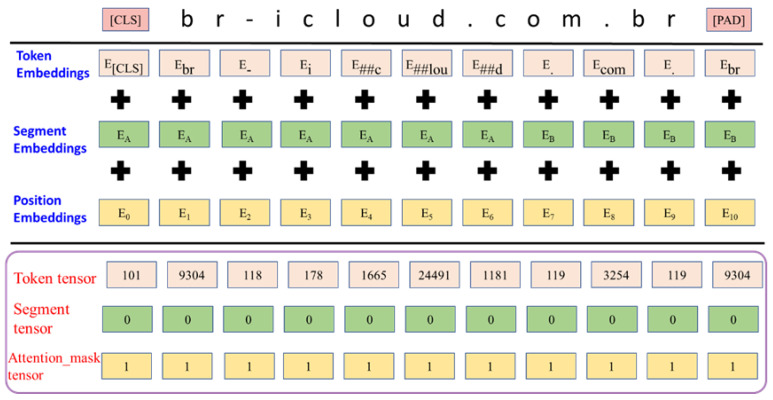
BERT input format.

Enhancing the performance of the pre-trained BERT model designed initially for natural language texts is essential when dealing with unnatural languages such as URLs. Transfer learning techniques were employed for this purpose. The choice of the pre-trained bert-base-cased model was motivated by its sensitivity to URL character cases. Input for the BERT model consists of three tensors, as depicted in Figure 5. The “tokens” tensor captures token embeddings, exemplified by the URL “br-icloud.com.br” being divided into 10 tokens. The initial {CLS} token marks the start of input and carries overall semantics. If the URL is short, {pad} tokens are appended at the end. The “segments” tensor distinguishes between sentences A (i.e., 0) and B (i.e., 1). However, given a single URL input, all values in the “segment” tensor are set to 0. Lastly, the “attention masks” tensor determines the scope of self-attention. In this study, the entire URL was treated as a single sentence, necessitating attention for all tokens. Consequently, all values in the “attention masks” tensor were set to 1.

**Figure 6:**
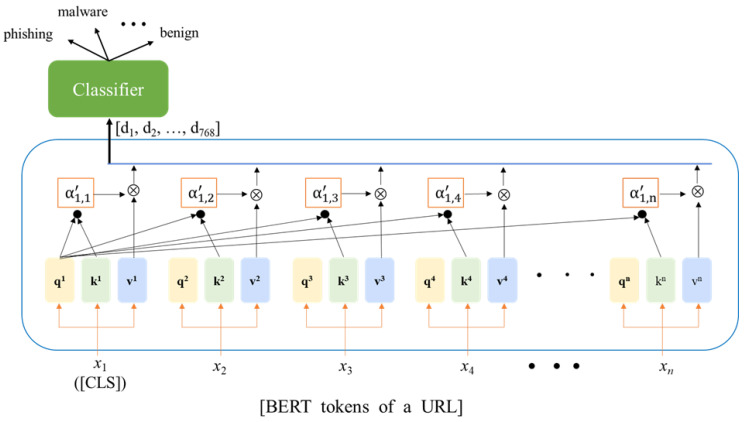
Illustration of self-attention, using token *x*_1_ as an example.

The output of the self-attention processing, representing the entire string—whether a URL string or a URL feature string—was encapsulated in the corresponding output {CLS}. A classifier was added to this {CLS} output for fine-tuning, as seen in Figure 6. In this study, the BertForSequenceClassification classifier was utilized. Once trained, the model could determine whether a given URL is normal or malicious. A brief introduction to the self-attention concept is provided below, while in-depth details about the BERT mechanism are available in [32].

Consider *X* = [*x*_1_, *x*_2_, …, *x_n_*] as the set of tokens within a URL, encompassing {CLS} as illustrated in Figure 6. Each input token *x_i_*, where 1 ≤ *i* ≤ *n*, has been transformed into a vector and subjected to multiplication with three weight matrices: *W^Q^*, *W^K^*, and *W^V^*, resulting in the corresponding triplets $\left(q_{i},k_{i},\mathrm{and}\;v_{i}\right)$, as indicated in Equations (1)–(3), respectively. The three matrices, *W^Q^*, *W^K^*, and *W^V^*, are derived through a learning process. The *q_i_* serves as the query, the *k_i_* serves as the key to be queried, and the *v_i_* represents the token’s information. For clarity, token *x*_1_ is employed to illustrate self-attention in Figure 6.

(1) $$x_{i}\cdot W^{Q}=q_{i}$$

(2) $$x_{i}\cdot W^{K}=k_{i}$$

(3) $$x_{i}\cdot W^{V}=v_{i}$$

In Figure 6, the *q*_1_ is utilized to query *k_j_*, where 1 ≤ *j* ≤ *n*, through an inner product operation as described in Equation (4). The value $\alpha_{1,j}$ represents the attention score of token *x*_1_ towards token *x_j_.* Subsequently, the Softmax function is applied to determine the proportions by which token *x*_1_ should be influenced by all tokens, as depicted in Equation (5). Ultimately, the output of token *x*_1_, denoted as *y*_1_, is obtained by summing the contributions from each token, as demonstrated in Equation (6).

(4) $$q_{1}\cdot k_{j}=\alpha_{1,j}$$

(5) $$\alpha_{1,j}^{\prime }=\frac{\exp\left(\alpha_{1,j}\right)}{\sum_{j}\exp\left(\alpha_{1,j}\right)}$$

(6) $$y_{1}=\sum_{i}\alpha_{1,j}^{\prime }v_{i}$$

Note that all the outputs of token *x_i_*, where 1 ≤ *i* ≤ *n*, can be calculated in parallel using Equation (7), where $Q=\left[q_{1},q_{2},\ldots ,q_{n}\right]$, $K=\left[k_{1},k_{2},\ldots ,k_{n}\right]$, $V=\left[v_{1},v_{2},\ldots ,v_{n}\right]$, and $\sqrt{d_{K}}$, denotes the dimensions of key *k*. The output of BERT is a 768-dimensional vector.

(7) $$\mathrm{self}\_\mathrm{attention}\left(Q,\;K,\;V\right)=\;\mathrm{softmax}\left(\frac{QK^{T}}{\sqrt{d_{k}\;}}\right)V$$

In summary, the model divides the URL into individual tokens and employs an attention mechanism to calculate contextual relationships among them. Each token is evaluated for its attention score in relation to others using Equations (1)–(6). Finally, Equation (7) is applied to extract the comprehensive semantic meaning of the entire URL, with the aim of determining whether the URL is malicious.

## 4. Experimental Results

This study conducted experiments using three distinct types of public datasets sourced from Kaggle [28], GitHub [29], and ISCX 2016 [30]. Specifically, Kaggle [28] exclusively contained URL strings, GitHub [29] provided URL features without accompanying URL strings, and ISCX 2016 [30] encompassed both URL strings and features. In assessing the efficacy of the proposed methods, various metrics, including accuracy, precision, and recall, were employed, as defined below:

$$\mathrm{Accuracy}=\frac{\mathrm{TP}+\mathrm{TN}}{\mathrm{TP}+\mathrm{TN}+\mathrm{FN}+\mathrm{FP}}$$

$$\mathrm{Recall}=\frac{\mathrm{TP}}{\mathrm{TP}+\mathrm{FN}}$$

$$\mathrm{Precision}=\frac{\mathrm{TP}}{\mathrm{TP}+\mathrm{FP}}$$

where, TP, TN, FN, and FP represent true positive, true negative, false negative, and false positive, respectively. This study utilized a computer equipped with an Intel Core i9 CPU with 64 GB memory and an NVIDIA RTX3070Ti GPU with 8 GB memory (ASUS WS750T). All the hyperparameters used in the experiments are detailed in Table 1.

### 4.1. Performance on Kaggle Dataset

**Figure 7:**
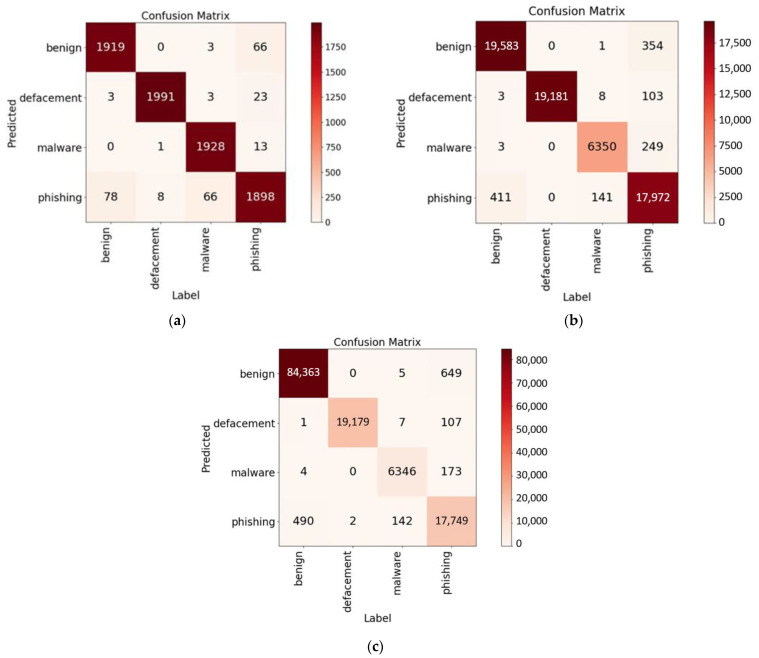
Different sizes of the Kaggle dataset used for experiments: (**a**) 40 thousand instances, (**b**) about 320 thousand instances, and (**c**) 646 thousand instances.

The Kaggle dataset [28] comprises four distinct URL types: benign, defacement, phishing, and malware. Among URL strings with lengths below 250 characters, the respective entry counts for these categories are approximately 424,000 for benign, 95,000 for defacement, 93,000 for phishing, and 32,000 for malware. Instances with exceptionally long lengths were infrequent; hence, they were excluded to reduce GPU memory usage and training duration. The dataset used in this study contained 99% of entries from the original dataset. For the initial experiment, a subset of ten thousand samples was randomly selected from each category, resulting in a total of 40 thousand samples. The results of this experiment are presented in Figure 7a, showing an accuracy rate of 96.70%. When expanding the sample size to min {one hundred thousand, actual number of entries} samples for each category, the results (shown in Figure 7b) indicate an improved accuracy rate of 98.02%. Finally, utilizing the entire dataset for experimentation, the outcomes (displayed in Figure 7c) exhibit an accuracy rate of 98.78%. In all three experiments, 80% of instances were allocated for training, while the remaining 20% were reserved for testing. Detailed results for these three experiments can be found in Table 2. A comparison with other related work is provided in Table 3.

### 4.2. Performance on GitHub Dataset

**Figure 8:**
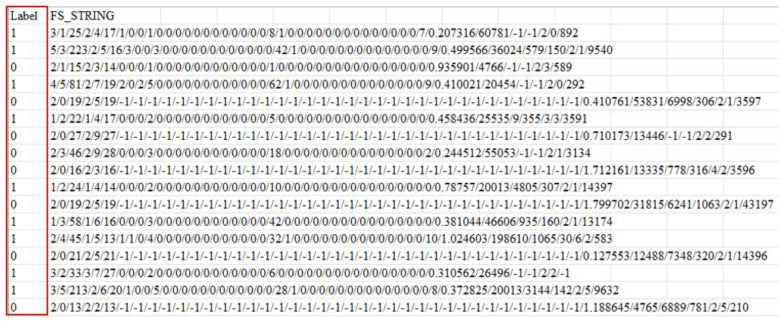
Feature strings of URLs after feature selection and concatenation.

**Figure 9:**
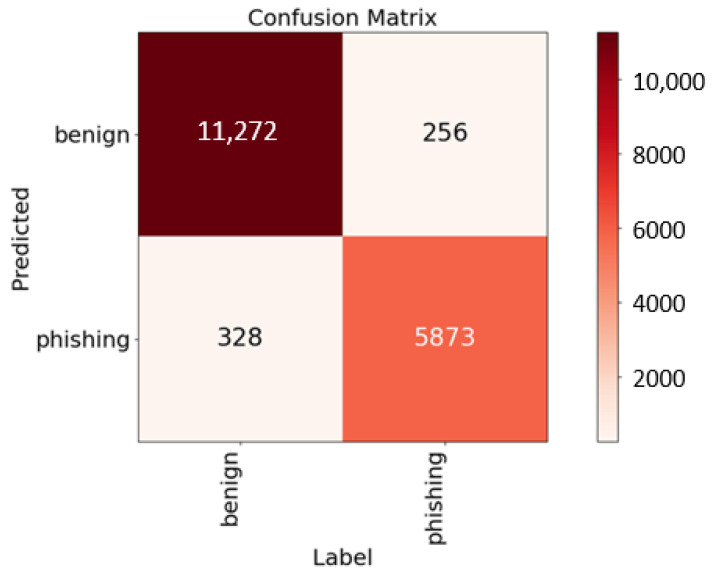
Confusion matrices for the GitHub dataset (*k* = 46).

The GitHub dataset [29] comprises approximately 88,000 entries, divided into two categories: 58,000 benign URLs and 30,000 phishing URLs. Unlike providing URL strings, this dataset furnished 111 features for each entry. In this study, the importance of each feature was computed using the random forest algorithm. From these, 46 essential features with an importance value exceeding 0.009 were selected. These features were then concatenated into a feature string, as illustrated in Figure 8. To preserve the integrity of individual features and facilitate self-attention within BERT tokens, the symbol “/” was employed for concatenation. With an 80–20% split for training and testing, the confusion matrix is depicted in Figure 9, where *k* = 46 denotes the number of selected features, showing accuracy, precision, and recall rates of 96.71%, 96.25%, and 96.50%, respectively.

**Figure 10:**
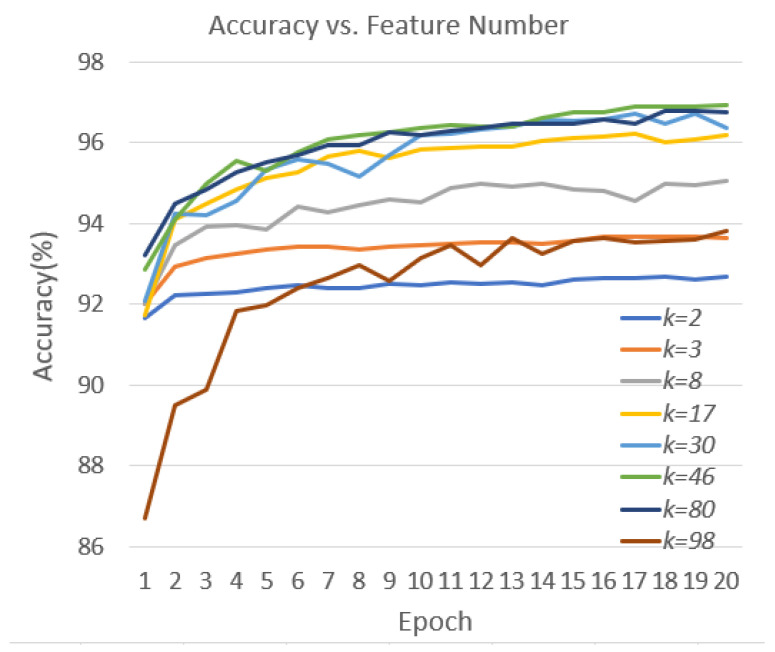
Learning curves of the proposed approach for different *k* values.

Table 4 outlines the performance metrics for different *k* values, while Figure 10 illustrates the learning curves of the validation accuracy for the model across various k values during training. To the best of our knowledge, there were no pertinent experimental results in the literature for this dataset, thus rendering direct comparisons unfeasible. Nonetheless, the proposed approach achieved a noteworthy accuracy of 96.71%, emphasizing its effectiveness in handling the dataset.

### 4.3. Performance on ISCX 2016 Dataset

**Figure 11:**
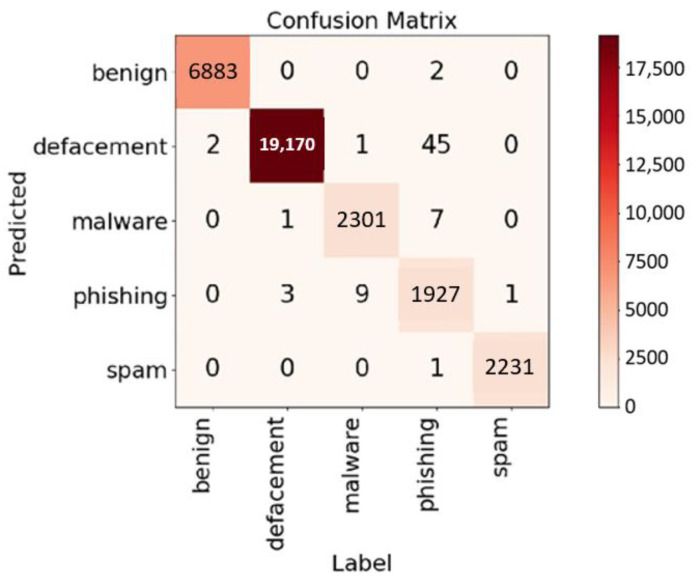
Confusion matrix for the multiclass classification on the ISCX 2016 dataset.

The ISCX 2016 dataset [30] comprises approximately 160,000 entries divided into five categories: around 35,000 benign URLs, 96,000 defacements, 11,000 malware, 11,000 spam, and 10,000 instances of phishing. The confusion matrix, with an 80–20% splitting for training and testing, is shown in Figure 11 as an epoch set to 30. The achieved accuracy, precision, and recall were 99.78%, 99.73%, and 99.34%, respectively. For the purpose of straightforward comparison with other research, a binary classification was also performed, wherein the four negative classes—defacements, malware, spam, and phishing—were collectively labeled as “malicious”. In this binary scenario, an impressive accuracy rate of 99.98% was attained. The comparative results are summarized in Table 5.

### 4.4. Extending to Other Domains

**Figure 12:**
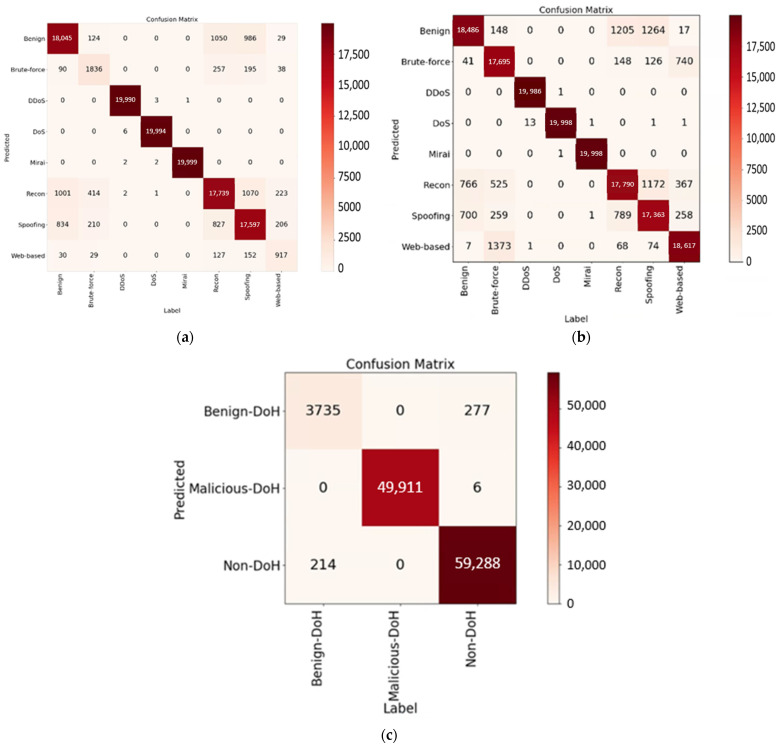
Confusion matrices for the IoT 2023 and DoHBrw 2020 datasets. (**a**) IoT 2023 dataset (original), (**b**) IoT 2023 dataset (augmented), and (**c**) DoHBrw 2020 (original).

The proposed feature string approach was further extended to include the detection of attacks targeting the Internet of Things (IoT) and attacks directed at DNS over HTTPS (DoH)—a protocol designed to enhance the security of DNS queries and responses. Two publicly available datasets, the “IoT Attack Dataset 2023” and the “DoHBrw 2020”, were utilized for this purpose and can be obtained from the website [33]. Detailed dataset information is also provided on the same website. The IoT dataset contains approximately 620,000 instances, categorized into eight classes, while the DoHBrw 2020 dataset consists of about 560,000 instances, falling into three classes. The confusion matrices for the two datasets, with an 80–20% split for training and testing, are presented in Figure 12a and Figure 12c, respectively. 

The performance metrics are summarized in Table 6. Notably, the IoT dataset exhibited class imbalance due to significantly fewer instances in the brute-force and web-based classes compared to others. In addressing this gap, the SMOTE algorithm [31] was employed to augment these two classes, ensuring a more balanced dataset. Additionally, experiments were conducted on the augmented IoT dataset. The confusion matrix is presented in Figure 12b, with corresponding numerical performance values included in Table 6.

### 4.5. URL Prediction Time

**Figure 13:**
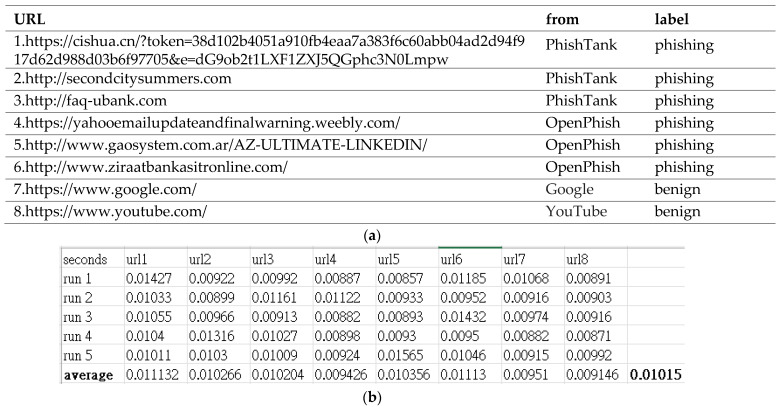
Average prediction time for one URL. (**a**) Tested URLs and (**b**) prediction time.

Moreover, the measurement of prediction times for real-time detection using the proposed approach was performed. Figure 13a illustrates the distribution of six malicious URLs and two benign URLs. The average prediction time per URL was approximately 0.010146 s, as shown in Figure 13b. These measurements were carried out on a desktop equipped with an Intel Core i9-3.50 GHz processor.

## 5. Conclusions and Future Work

This study presents a BERT-based approach for non-natural language processing tasks, with a specific focus on identifying malicious URLs. Through extensive experiments carried out on three distinct public datasets (Kaggle, GitHub, and ISCX 2016), the effectiveness of the proposed model has been demonstrated. In comparison to previous research, the proposed system outperforms in terms of accuracy. In the multi-classification experiments conducted on the Kaggle dataset, the achieved accuracy was 98.78%. For the GitHub dataset, which provides only features without corresponding URL strings, the proposed model exhibited an accuracy of 96.71%. In the ISCX 2016 dataset experiments, the model displayed remarkable accuracy rates of 99.98% in binary classification and 99.78% in multi-classification tasks. Furthermore, two datasets from different domains concerning IoT and DNS over HTTPS were incorporated into the study to demonstrate the versatility of the proposed system. Moreover, the proposed pre-trained model can make decisions on tested URLs quickly, making the system suitable for real-time detection deployment. Indeed, the BERT-based approach demonstrates superior performance when compared to other methods in experiments with existing URL datasets. However, its effectiveness in detecting zero-day malicious URL attacks, including newly registered URLs or benign web servers that have turned malicious due to infections, remains uncertain. In the future, we aspire to conduct further investigations into these related issues.

## Figures and Tables

**Table 1:** Hyperparameters used for training the proposed model.

**attention probs dropout prob**	**hidden act**	**hidden dropout prob**	**initializer range**	**intermediate size**	**type vocab size**	**max seq length**	**BERT model**	**num train epochs**
0.1	gelu	0.1	0.02	3072	2	128	bert-base-cased	10/20/30
**max position embeddings**	**hidden size**	**num attention heads**	**layer norm eps**	**num hidden layers**	**learning rate**	**train batch size**	**vocab size**	
512	768	12	1 × 10^−12^	12	1 × 10^−5^	16	28,996	

**Table 2:** Kaggle dataset for multiclass classification.

Samples for Each Category	#URLs	Accuracy (%)	Precision (%)	Recall (%)
10,000	40,000	96.70	96.74	97.73
Min {100,000, actual entry number}	about 320,000	98.02	97.71	97.96
All	646,083	98.78	99.12	98.02

**Table 3:** Comparison with the literature using the Kaggle dataset.

Research	Approach	Binary/Multi Classification	Accuracy (%)
Alsaedi et al. [21] (2022)	Ensemble features + Classifiers (DT)	Binary	95.70
	Ensemble features + Classifiers (RF)	Binary	96.80
	Ensemble features + Classifiers (CNN)	Binary	94.70
	Ensemble features + Classifiers (RF with feature selection)	Binary	96.80
	Ensemble features + Classifiers (RF with feature selection and grid search best-found hyperparameters)	Binary	97.25
This study	Fine-tuning BERT + Classifier	Multi	98.78

**Table 4:** Performance of the different number of features (i.e., *k*) selected on the GitHub dataset.

*k* (#Features)	Accuracy (%)	Precision (%)	Recall (%)
2	92.64	91.33	92.99
3	93.36	92.38	93.09
8	94.69	94.44	93.76
17	95.84	95.64	95.14
30	95.93	95.83	95.14
46	96.71	96.25	96.50
80	96.53	96.17	96.15
86	96.42	96.48	95.70

**Table 5:** Comparison with other research on the ISCX 2016 dataset.

Research	Binary/Multi-Classification	Accuracy (%)	Approach
Saleem et al. [6] (2021)	Binary	99	Feature extraction + RF
Gupta et al. [5] (2021)	Binary (phish only)	99.57	Feature extraction + RF
Bozkir et al. [10] (2023)	Binary (phish only)	99.82	n-gram + CNN_BiLSTM + Attention
Wang and Chen [23] (2022)	Binary (phish only)	99.77	CNN1D + Transformer
	Binary (phish only)	98.67	CNN-MHSA
	Binary (phish only)	99.57	BiLSTM
This study	Binary	99.98	Fine-tunned BERT + Classifier
	Multi	99.78	

**Table 6:** Expanding the proposed method to other domains.

Dataset		Accuracy (%)	Precision (%)	Recall (%)
IoT Attack Dataset 2023	original	93.62	89.29	87.75
	augmented by SMOTE [31]	93.71	93.77	93.71
DoHBrw 2020 (original)		99.56	97.57	98.04

## Data Availability

All datasets used in this study are publicly available.
